# Evaluation of Intravenous Fosfomycin Disodium Dosing Regimens in Critically Ill Patients for Treatment of Carbapenem-Resistant Enterobacterales Infections Using Monte Carlo Simulation

**DOI:** 10.3390/antibiotics9090615

**Published:** 2020-09-18

**Authors:** Pannee Leelawattanachai, Thitima Wattanavijitkul, Taniya Paiboonvong, Rongpong Plongla, Tanittha Chatsuwan, Sang Usayaporn, Wichit Nosoongnoen, Preecha Montakantikul

**Affiliations:** 1 Division of Clinical Pharmacy, Department of Pharmacy, Faculty of Pharmacy, Mahidol University, Bangkok 10400, Thailand; pannee@nmu.ac.th (P.L.); wichit.nos@mahidol.ac.th (W.N.); 2 Department of Pharmacy, Faculty of Medicine Vajira Hospital, Navamindradhiraj University, Bangkok 10300, Thailand; 3 Department of Pharmacy Practice, Faculty of Pharmaceutical Sciences, Chulalongkorn University, Pathumwan, Bangkok 10330, Thailand; thitima.w@pharm.chula.ac.th (T.W.); sang.u@pharm.chula.ac.th (S.U.); 4 Department of Pharmacy Practice, College of Pharmacy, Rangsit University, Pathum Thani 12000, Thailand; taniya.p@rsu.ac.th; 5 Division of Infectious Diseases, Department of Medicine, Faculty of Medicine, Chulalongkorn University, and King Chulalongkorn Memorial Hospital, Thai Red Cross Society, Bangkok 10330, Thailand; rongpong.p@chula.ac.th; 6 Antimicrobial Resistance and Stewardship Research Unit, Chulalongkorn University, Bangkok 10330, Thailand; tanittha.c@chula.ac.th; 7 Department of Microbiology, Faculty of Medicine, Chulalongkorn University, Bangkok 10330, Thailand

**Keywords:** intravenous fosfomycin disodium, carbapenem-resistant Enterobacterales, pharmacokinetics/pharmacodynamics, dosing regimens, critically ill patients

## Abstract

There are limited intravenous fosfomycin disodium (IVFOS) dosing regimens to treat carbapenem-resistant Enterobacterales (CRE) infections. This study aimed to use Monte Carlo simulation (MCS) for evaluation of IVFOS dosing regimens in critically ill patients with CRE infections. The dosing regimens in critically ill patients with various creatinine clearance were evaluated with MCS using minimum inhibitory concentration (MIC) distributions of fosfomycin against CRE clinical isolates in Thailand and the 24 h area under the plasma drug concentration–time curve over the minimum inhibitory concentration (AUC_0-24_/MIC) of ≥21.5 to be a target for IVFOS. The achieved goal of the probability of target attainment (PTA) and a cumulative fraction of response (CFR) were ≥90%. A total of 129 non-duplicated CRE clinical isolates had MIC distributions from 0.38 to >1024 mg/L. IVFOS 8 g every 8 h, 1 h, or 4 h infusion, could achieve approximately 90% PTA of AUC_0-24_/MIC target to treat CRE infections with MICs ≤ 128 mg/L. According to PTA target, an IVFOS daily dose to treat carbapenem-resistant *Escherichia coli* based on Clinical Laboratory Standards Institute (CLSI) breakpoints for urinary tract infections and one to treatment for CRE infections based on the European Committee on Antimicrobial Susceptibility Testing (EUCAST) breakpoints were 16 g/day and 8 g/day, respectively. All dosing regimens of IVFOS against CRE achieved CFR ≤ 70%. This study proposes the IVFOS dosing regimens based on CLSI and EUCAST breakpoints for the treatment of CRE infections. However, further clinical studies are needed to confirm the results of these findings.

## 1. Introduction

Carbapenem-resistant Enterobacterales (CRE) infections cause public health problems worldwide with limited antibiotic treatments. Fosfomycin is one of the mainstay therapies to treat CRE infections [1,2]. Fosfomycin shows broad-spectrum antimicrobial activity and inhibits cell wall synthesis of bacteria. Intravenous fosfomycin disodium (IVFOS) has been used in combination with other agents to treat multidrug-resistant bacterial infections [3,4]. This formulation is available in many countries outside the United States. The dosage recommendations of IVFOS are 12 to 24 g/day, according to the European Committee on Antimicrobial Susceptibility Testing (EUCAST) guidelines and manufacturer’s recommendations [5,6]. However, this information is insufficient for guiding treatments for CRE infections.

In addition, the physiochemical properties were concerned. IVFOS is a hydrophilic drug, with a volume distribution of around 20 to 40 L; it is negligibly protein-bound, and results in almost total renal elimination [7,8]. Results from several population pharmacokinetic studies showed that fosfomycin was a significant variable of plasma concentration, and large interindividual with intraindividual pharmacokinetic variabilities [9,10,11]. Therefore, small changes in critically ill patients may affect fosfomycin plasma concentration. A previous population pharmacokinetic study of IVFOS in critically ill patients showed inadequate dosing for bacterial eradication [11]. In addition, IVFOS demonstrated that 0.5 h to 1 h infusion was more associated with hypokalemia than 4 h infusion [12]. The pharmacokinetic/pharmacodynamic (PK/PD) index is necessary for evaluating the efficacy and safety of IVFOS dosing regimens. Several pharmacodynamic studies of fosfomycin against CRE showed that the best PK/PD index of fosfomycin was the 24 h area under the plasma drug concentration–time curve over the minimum inhibitory concentration (AUC_0-24_/MIC) [13,14,15].

Monte Carlo simulation was used to determine dosage regimens and assist in the selection of appropriate empirical therapies. This simulation was able to relate the pharmacokinetic parameter, and pharmacodynamic parameter with minimum inhibitory concentration (MIC) for predicting the probability of treatment outcome [16,17]. However, there is currently no Monte Carlo simulation study using the AUC_0-24_/MIC target of fosfomycin against CRE to evaluate the dosing regimens of IVFOS in critically ill patients. Therefore, the purpose of this study was to use Monte Carlo simulation to evaluate the dosing regimens of IVFOS in critically ill patients to treat infections caused by CRE.

## 2. Results

### 2.1. MIC Distributions

A total of 129 non-duplicated CRE retrospective clinical isolates were collected during the three years study period. Carbapenem-resistant *Klebsiella pneumoniae* (CR-KP) was the most frequent isolate. The most common sources of infection were the bloodstream (38.76%) and urine (20.93%). The fosfomycin susceptibility and distributions of fosfomycin MICs against CRE clinical isolates are presented in Table 1.

### 2.2. Pharmacokinetic/Pharmacodynamic Simulations

**Figure 1:**
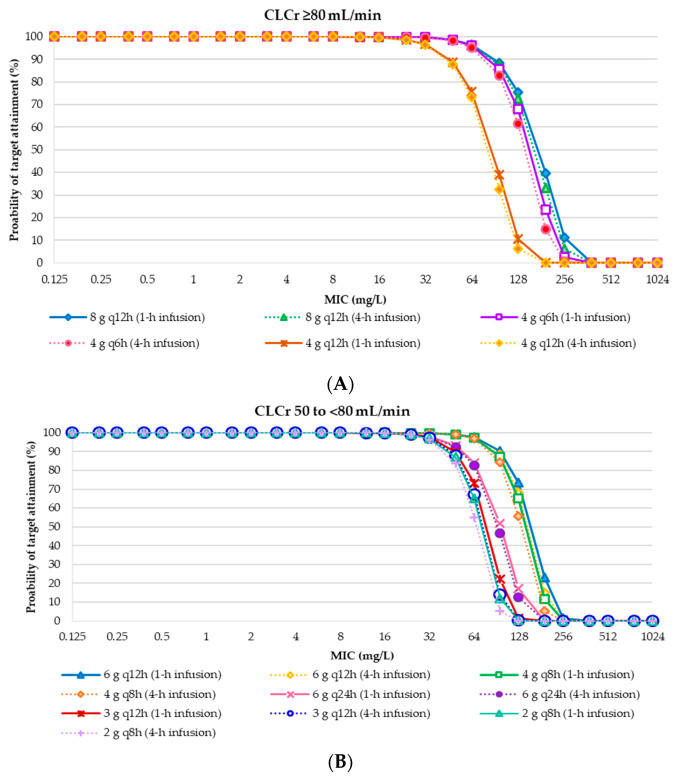

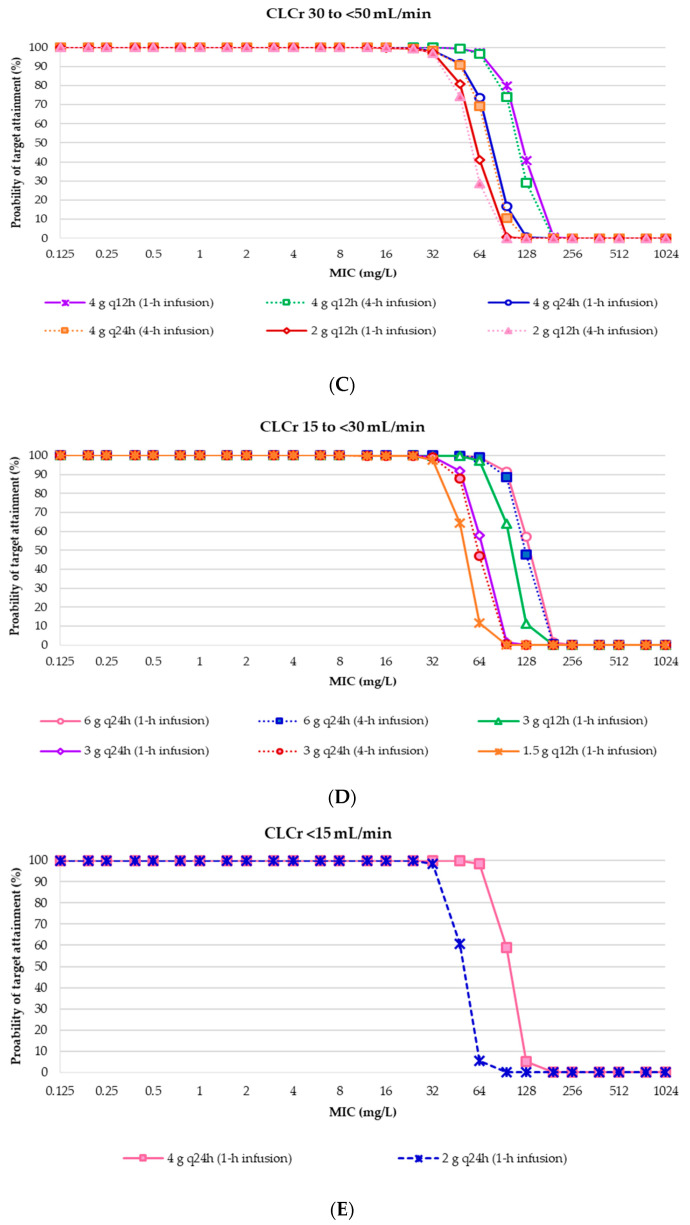
Probability of target attainment of intravenous fosfomycin disodium dosing regimens achieving AUC_0-24_/MIC at least 21.5 in critically ill patients with a weight of 50 kg and various degrees of renal function (CLCr). (**A**) CLCr ≥ 80 mL/min; (**B**) CLCr 50 to <80 mL/min; (**C**) CLCr 30 to <50 mL/min; (**D**) CLCr 15 to <30 mL/min; (**E**) CLCr <15 mL/min. Abbreviation: CLCr, creatinine clearance; q6h, every 6 h; q8h, every 8 h; q12h, every 12 h; q24h, every 24 h.

**Figure 2:**
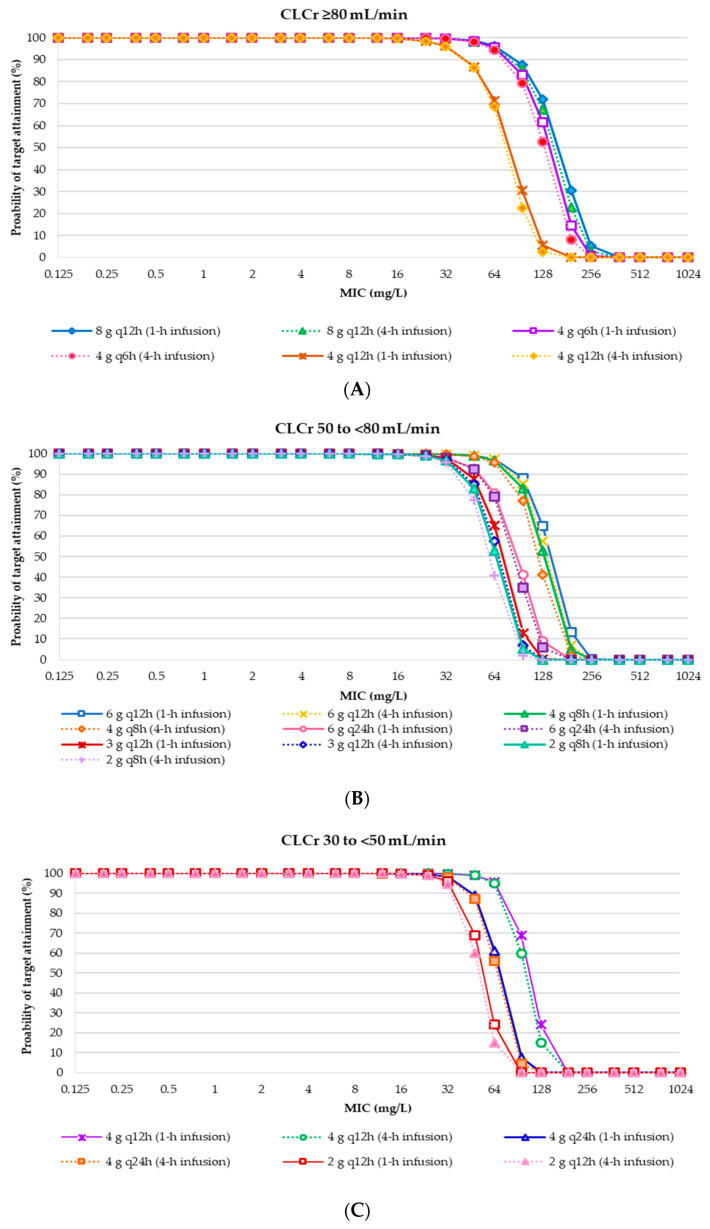

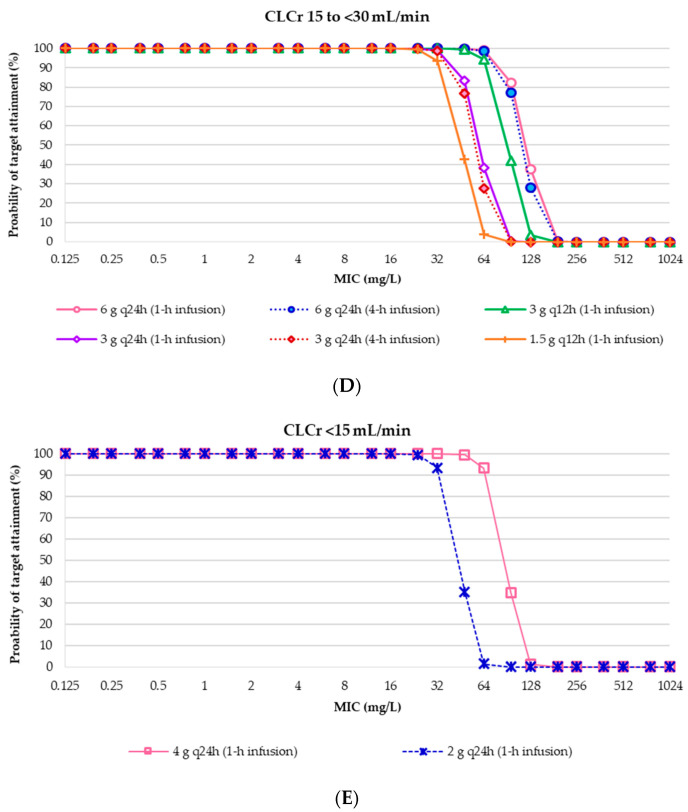
Probability of target attainment of intravenous fosfomycin disodium dosing regimens achieving AUC_0-24_/MIC at least 21.5 in critically ill patients with a weight of 70 kg and various degrees of renal function (CLCr). (**A**) CLCr ≥ 80 mL/min; (**B**) CLCr 50 to <80 mL/min; (**C**) CLCr 30 to <50 mL/min; (**D**) CLCr 15 to <30 mL/min; (**E**) CLCr < 15 mL/min. Abbreviation: CLCr, creatinine clearance; q6h, every 6 h; q 8 h, every 8 h; q 12 h, every 12 h; q 24 h, every 24 h.

The probability of AUC_0-24_/MIC ratio of ≥ 21.5 of target attainment for each IVFOS dosing regimen in critically ill patients with weights of 50 kg and 70 kg, and various degrees of renal functions (Tables S1 and S2). The results showed that the IVFOS dosing regimens of 24 g/day achieved approximately 90% probability of target attainment (PTA) for MICs ≤ 128 mg/L in critically ill patients with creatinine clearance (CLCr) ≥ 50 mL/min. All dosing regimens of IVFOS in critically ill patients with various degrees of renal function achieved a PTA of >90% when MICs ≤ 32 mg/L, in accordance with the EUCAST susceptibility breakpoints [18]. The PTA ranged from 1.46% to 99.99% were observed for MIC of 64 mg/L, which is the susceptibility breakpoints of Clinical and Laboratory Standards Institute (CLSI) [19]. These results were used to suggest the daily doses for individual patients with various degrees of renal function, according to the CLSI and EUCAST breakpoints [18,19], is presented in Figure 1 and Figure 2, and Table 2. All dosing regimens of IVFOS achieved < 90% cumulative fraction of response (CFR) against MIC distributions of clinical isolates (Tables S1 and S2).

## 3. Discussion

Our study found that 89.92% of CRE isolates were CR-KP. Similar to a previous study, CR-KP was the most common cause of CRE infections [20]. Our study showed that the fosfomycin MICs of carbapenem-resistant *Escherichia coli* (CR-EC) clinical isolates were lower than that of CR-KP clinical isolates. This finding could be explained by the mechanisms of Enterobacterales resistant to fosfomycin, which was associated with the gene of the fosfomycin-modifying enzyme, *fosA3*. The *fosA3* gene has been found in the plasmid of CTX-M-producing *Escherichia coli* and KPC-producing *Klebsiella pneumoniae* isolates [21,22]. Therefore, the variation of the fosfomycin MICs may depend on the species of bacteria [23]. However, the numbers of CR-EC clinical isolates in our study were insufficient to explain the differences in the fosfomycin MIC distributions. Additionally, our study did not perform the molecular mechanisms of CRE and fosfomycin-resistant Enterobacterales. These genotypic descriptions are required for further studies.

Fosfomycin susceptibility testing of CRE clinical isolates in our study was interpreted, according to both the CLSI and EUCAST breakpoints [18,19]. However, CLSI breakpoints were used to interpret fosfomycin susceptibility of urinary isolates of *Escherichia coli* only [19]. Our results and the report of the National Antimicrobial Resistance Surveillance Center, Thailand (NARST) [24] demonstrated that 100% and 97.9% of *Escherichia coli* clinical urinary isolates were susceptible to fosfomycin, respectively. Our study also showed that the percentages of fosfomycin-susceptible CR-KP were 40.52%. Whereas, previous studies demonstrated that 89.7% of CR-KP was susceptible to fosfomycin [25]. This result may be explained by the epidemiology of carbapenemase producers and mechanisms of fosfomycin-resistant Enterobacterales. Our study collected clinical isolates from a university hospital in Thailand, which is associated with the epidemiology of the carbapenemase producers, including NDM-1-producers and OXA-48-producers [26,27,28,29,30]. In contrast, the epidemiology of carbapenemase producers of a previous study was dominated by KPC producers [25]. In addition, a previous study showed that fosfomycin-resistant in KPC-producing *Klebsiella pneumoniae* isolates was associated with the *fosA3* gene [22]. While there is currently no report of OXA-48-producers or NDM-1-producing, *Klebsiella pneumoniae* isolates were associated with the *fosA* gene. This may be caused by other mechanisms. The result of our simulations demonstrated that IVFOS dosing regimens in critically ill patients with weights of 50 kg and 70 kg and CLCr 50 to ≥80 mL/min at 24 g/day given every 8 h, 1 h, or 4 h infusion achieved approximately 90% PTA when fosfomycin MICs ≤ 128 mg/L (Tables S1 and S2). This dosing regimen is the maximum recommended dose, according to the EUCAST guideline and manufacturer’s recommendations [5,6]. This finding differs from previous studies [9,31,32,33]. Albiero et al. demonstrated that IVFOS 24 g/day given every 6 h and 8 h, 0.5 h and 3 h infusion also achieved 90% PTA at MICs ≤ 16 mg/L [31]. Rodríguez-Gascón et al. showed that IVFOS 24 g/day given every 6 h, 0.5 h infusion, and 24 g/day given every 8 h, 0.5 h, and 6 h infusion also achieved 90% PTA at MICs ≤ 64 mg/L [32]. Bhavnani et al. demonstrated that IVFOS 18 g/day given every 8 h achieved 90% PTA at MICs ≤ 64 mg/L [33]. Merino-Bohórquez et al. showed that IVFOS 24 g/day given every 8 h, 1 h infusion, and 16 g/day given every 6 h, 1 h infusion also achieved > 90% PTA at MICs ≤ 32 mg/L [9]. These results could be explained by the differences in pharmacokinetic parameters and PK/PD index for Monte Carlo simulation in each study. Our study used pharmacokinetic parameters in critically ill patients similar to those of Albiero et al. and Rodríguez-Gascón et al. [31,32]. In contrast, Bhavnani et al. and Merino-Bohórquez et al. also used pharmacokinetic parameters in non-critically ill patients [9,33]. The PK/PD index in our study provided an AUC_0-24_/MIC target for fosfomycin against NDM–1–producing *Klebsiella pneumoniae* that differed from that of Albiero et al. (70% *f* T > MIC of fosfomycin against *Listeria monocytogenes)*, or Rodríguez-Gascón et al., Bhavnani et al. and Merino-Bohórquez et al. (AUC_0-24_/MIC of fosfomycin against Enterobacterales) [9,31,32,33]. Moreover, our study found that none of the IVFOS dosing regimens achieved the PTA of ≥ 90% at the MICs ≥ 192 mg/L. This result supports the use of IVFOS in combination with other agents for the treatment of CRE infections with high fosfomycin MICs.

For critically ill patients with CLCr 30 to <50 mL/min and weights of 50 kg and 70 kg, the result showed that IVFOS 16 g/day given every 12 h, 1 h infusion achieved > 90% PTA at MICs ≤ 128 mg/L. This is similar to a previous study by Bhavnani et al., which demonstrated that IVFOS 18 g/day given every 8 h achieved > 90% PTA at MICs ≤ 128 mg/L [33]. This could be explained by the renal function, which was a significant covariate that affects fosfomycin clearance.

The result in critically ill patients with weights of 50 kg and 70 kg, and CLCr 15 to <30 mL/min showed that IVFOS 12 g/day given every 8 h, 1 h infusion achieved > 90% PTA at MICs ≤ 96 mg/L. The result is different from the result of Bhavnani et al., who showed that IVFOS 18 g/day given every 8 h achieved > 90% PTA at MICs ≤ 64 mg/L [33]. In addition, critically ill patients with weights of 50 kg and 70 kg, and CLCr <15 mL/min without hemodialysis, IVFOS 6 g/day given every 12 h and 24 h, 1 h and 4 h infusion also achieved > 90% PTA at MICs ≤ 64 mg/L. This dosing regimen was higher than the maximum dosage recommended by the manufacturer [6], and there were no previous studies in humans. Therefore, the safety of the higher dose should be monitored.

Interestingly, all dosing regimens in critically ill patients with a weight of 50 kg achieved a higher PTA than critically ill patients with a weight of 70 kg. This finding may be explained by the final model of pharmacokinetic parameters in which weight was a significant covariate of volume of central compartment (Vc). However, the recommended dosage was similar.

Electrolyte imbalance, including hypernatremia and hypokalemia, is a safety concern in patients receiving IVFOS. A previous observational study demonstrated that IVFOS 12 g/day given every 8 h, 0.5 h to 1 h infusion caused greater hypokalemia than 4 h infusion (*p* = 0.007) [12]. Thus, our study designed an infusion rate of 1 h and 4 h for simulation. Interestingly, our results demonstrated that the infusion rate of all IVFOS dosing regimens of 1 h and 4 h had no significant effect on achieving PTA target. Therefore, the prolonged infusion of 4 h could be an appropriate alternative regimen to reduce the electrolyte imbalance from IVFOS.

Our study suggested new dosing regimens are needed for IVFOS in patients with various degrees of renal function, according to the CLSI and EUCAST for fosfomycin susceptibility breakpoints (Table 2). The fosfomycin susceptibility breakpoints of CLSI (MICs ≤ 64 mg/L) were applied to *Escherichia coli* urinary tract infections [19]. Therefore, new IVFOS dosing regimens, according to the CLSI breakpoints, were applied to treat urinary tract infections caused by CR-EC. This finding is consistent with the recommended dosage regimens from the manufacturer [6]. However, dosing regimens for patients with CLCr 40 to 80 mL/min were not recommended by the manufacturer [6]. This result suggests the need for new dosing regimens in these patients. In addition, the result showed that 6 g/day was recommended, but intervals for new dosing regimens and the manufacturer’s recommendations [6] in patients with CLCr 15 to <30 mL/min were every 12 or 24 h, and every 8 or 12 h, respectively. This finding may be explained by the relationship of dosing regimens, PTA, and the fosfomycin MICs. Our study demonstrated that IVFOS 6 g/day given every 8 h, 1 h infusion achieved PTA of 91.43% and 82.48% in patients with weights of 50 kg and 70 kg, respectively (data not shown).

In addition, the fosfomycin susceptibility breakpoint of EUCAST for Enterobacterales was MICs ≤ 32 mg/L [18]. The new IVFOS dosing regimens, according to EUCAST breakpoints, were applied to treat CRE infections. This result suggested that the initial dose of IVFOS should be 8 g/day given every 12 h, 1 h, or 4 h infusion, which was lower than the recommendation of EUCAST and manufacturer (12 to 24 g/day) [5,6]. This finding might be explained by the method used to determine the dosing regimens. In our study, AUC_0-24_/MIC of ≥ 21.5 as a PK/PD index, pharmacokinetic parameters of critically ill patients, and MIC breakpoints were applied. In contrast, the EUCAST guideline did not use Monte Carlo simulation to determine dosing regimens [5]. Moreover, the manufacturer did not have a recommended dose in patients with CLCr 40 to 80 mL/min [6]. Whereas, our findings suggested new dosing regimens in these patients, according to the EUCAST breakpoints (Table 2).

Our study provided the MIC distributions of fosfomycin against CRE and CR-KP from King Chulalongkorn Memorial Hospital, Thailand, to determine the CFR of IVFOS dosing regimens. The result showed that none of the IVFOS dosing regimens achieved the CFR target of 90%. This finding may be explained by the MIC_90_ of fosfomycin against CRE and CR-KP were over 1024 mg/L. This result is consistent with previous studies [34,35]. The previous studies demonstrated that the combination therapy of IVFOS with other antibiotics was associated with a reduction of mortality in critically ill patients with CRE infections [34,35]. However, some previous studies showed that IVFOS monotherapy was associated with high clinical cure rate (100%) and microbiological eradication (56%) in patients with acute pyelonephritis caused by CRE [36,37]. The difference might be explained by the epidemiology, susceptibility, and MIC distributions of fosfomycin against CRE. MICs of fosfomycin against CRE in our study (MIC_90_ > 1024 mg/L) were higher than that of the previous studies (MICs 0.5 to >512 mg/L) [36,37]. Additionally, our study showed that most of Enterobacterales clinical isolates were *Klebsiella pneumoniae*. CFR of IVFOS dosing regimens might be more associated with CR-KP infection than CRE infection. However, a large number of CRE clinical isolates were required to confirm these findings. Our study suggested that IVFOS should be used in combination with other agents for the empirical treatment of infection caused by CRE or CR-KP with high fosfomycin MICs, e.g., MICs > 1024 mg/mL.

Our study had several limitations. First, a loading dose was not simulated in our study. However, the steady-state of pharmacokinetic parameters was applied in our simulation. Therefore, our study suggests daily doses for maintenance dosing regimens. Second, fosfomycin susceptibility testing was conducted by the Epsilometer test (Etest). The CLSI and EUCAST recommended agar dilution, which was a standard method for testing the fosfomycin susceptibility [18,19]. Previous studies demonstrated that Etest and agar dilution had a variable correlation [25]. Therefore, this method may affect the interpretation of fosfomycin MICs. Finally, our study had small numbers of CRE clinical isolates, which may be insufficient to present fosfomycin MIC distributions resulting in reduced accuracy of CFR prediction.

## 4. Materials and Methods

### 4.1. Pharmacokinetic Parameters

The two-compartment pharmacokinetic model was used to determine the concentration-time profiles of each dosing regimen of IVFOS at a steady-state. Pharmacokinetic parameters from a previous population pharmacokinetic study of IVFOS in adult critically ill patients were applied [11]. Briefly, the final model included total clearance (CL) = 4.13 × (CLCr/90), Vc = 26.5 × (weight/70)^0.75^, volume of peripheral compartment was 22.3 L, and intercompartmental clearance was 19.8 L/h. The important covariates of the model were CLCr calculated by Cockcroft-Gault Equation and the bodyweight used to evaluate the IVFOS dosing regimens in this study. The population bodyweights used in this study were 50 kg and 70 kg, according to female and male representatives, respectively. Interindividual variation of the model, consisting of CL and Vc, were considered log-normal distributions with coefficients of variation (CV) of 91.9% and 39%, respectively [11].

### 4.2. Microbiology

One hundred and twenty-nine CRE clinical isolates were tested for fosfomycin MICs. All isolates were identified from all clinical specimens of patients at King Chulalongkorn Memorial Hospital, Thailand, from January 2016 to December 2018. CRE was defined as Enterobacterales resistant to ertapenem, imipenem, meropenem, or doripenem, according to the Centers for Disease Control and Prevention (CDC) criteria [38]. Fosfomycin MICs were tested using the Etest (BioMérieux, Durham, NC, USA) according to the manufacturer’s instructions in the hospital’s clinical laboratories. Fosfomycin MICs were interpreted using the current MIC breakpoints, according to the CLSI and EUCAST [18,19].

The study protocol was approved by the Institutional Review Board, Faculty of Dentistry, and Faculty of Pharmacy, Mahidol University, Thailand (No.MU-DT/PY-IRB 2019/058.3008).

### 4.3. Pharmacodynamic Parameters

Pharmacodynamic parameters of fosfomycin were obtained from a previously published study that used the neutropenic murine model [14]. The study demonstrated that the PK/PD index of fosfomycin against NDM–1–producing *Klebsiella pneumoniae* for the best prediction of response was the AUC_0-24_/MIC ratio of ≥21.5 associated with 1-log_10_ CFU reduction of bacterial burden [14]. This PK/PD index was used as an optimal target to evaluate each dosing regimen of IVFOS. Protein binding of fosfomycin was negligible.

### 4.4. Intravenous Fosfomycin Disodium Dosing Regimens

IVFOS dosing regimens for simulation were derived from our own determination and modification of IVFOS manufacturer’s recommendations (Nordic Pharma UK Limited, Germany) [6]. The dosing regimens were simulated in critically ill patients with weights of 50 kg and 70 kg, and various degrees of renal functions. The range of CLCr for simulations was from 5 to 90 mL/min, which was increased by 5 to 10 mL/min. The following dosing regimens were evaluated from 2 to 24 g/day given every 6 to 24 h with short infusions of 1 h and prolonged infusions of 4 h, as shown in Table 3. The simulation of each dosing regimen was based on each CLCr (Table 3), such as IVFOS 24 g/day given every 8 h, 1 h, and 4 h infusion simulated based on CLCr 50 to ≥80 mg/min.

### 4.5. Monte Carlo Simulation

Monte Carlo simulation of 10,000 subjects were performed using Crystal ball^®^ software (Oracle Corporation, version 11.1.2.4, Redwood Shores, CA, USA). For each IVFOS dosing regimen of each CLCr (Table 3), the following process was titrated from the 1st to the 10,000th patients. Monte Carlo simulation used the population pharmacokinetic model [11] to determine the actual individual exposures by calculating the 24 h area under the plasma drug concentration–time curve (AUC_0-24_) at various MIC values for every 10,000 subjects, based on individual pharmacokinetic parameter estimated. The result of the Monte Carlo simulation was presented as the PTA for each dosing regimen of IVFOS. The PTA was defined as the percentage of subjects who achieved the PK/PD index for each IVFOS dosing regimen against the bacterial population. The PK/PD index was selected based on pharmacodynamic properties of fosfomycin against NDM–1–producing *Klebsiella pneumoniae* as the AUC_0-24_/MIC ratio of ≥ 21.5 [14]. The CFR was calculated from PTA based on the MIC distributions of fosfomycin against CRE from King Chulalongkorn Memorial Hospital, Thailand. The PTA and CFR results of at least 90% were selected to evaluate each dosing regimen.

## 5. Conclusions

Our study suggested dosing regimens for IVFOS based on CLSI and EUCAST breakpoints. These results may be an alternative regimen to treat CRE infections in critically ill patients with various degrees of renal function. For CRE infections in Thailand, our study demonstrated that IVFOS could be an inadequate empirical monotherapy, and requires combination with other agents. However, further clinical studies are needed to confirm the results of these findings.

## Figures and Tables

**Table 1:** The minimum inhibitory concentrations (MICs) of fosfomycin for 129 clinical isolates of carbapenem-resistant Enterobacterales.

Carbapenem-Resistant Enterobacterales Isolates	Number of Isolates	Fosfomycin MICs (mg/L)			% of Isolates	
					CLSI ^a^	EUCAST ^b^
		MIC_50_	MIC_90_	MICs Range	S/I/R	S/R
*Klebsiella pneumoniae*	116	64	>1024	0.38 to >1024	-	40.52/59.48
*Escherichia coli*	12	NA ^c^	NA ^c^	1 to 16	100/0/0	100/0
*Enterobacter cloacae*	1	NA ^c^	NA ^c^	12	-	100/0
All isolates	129	48	>1024	0.38 to >1024	-	46.51/53.49

MICs = minimum inhibitory concentrations; MIC_50_ = MIC for 50% of clinical isolates; MIC_90_ = MIC for 90% of clinical isolates; CLSI = Clinical and Laboratory Standards Institute; EUCAST = European Committee on Antimicrobial Susceptibility Testing; - = no breakpoint criteria for interpretation; NA = not available data. ^a^ CLSI breakpoints for *Escherichia coli* urinary isolates, MIC ≤ 64 mg/L as susceptible (S), MIC =128 mg/L as intermediate (I), and MIC ≥ 256 mg/L as resistant (R). ^b^ EUCAST breakpoints for Enterobacterales spp. isolates, MIC ≤ 32 mg/L as susceptible (S), and MIC > 32 mg/L as resistant (R). ^c^ the small numbers of clinical isolates.

**Table 2:** Daily dosing suggestions for intravenous fosfomycin disodium to treat carbapenem-resistant Enterobacterales infections, according to CLSI and EUCAST breakpoints.

CLCr (mL/min) ^a^	Daily Doses Suggestion		Dosage Adjustments
	CLSI Breakpoints ^b^	EUCAST Breakpoints ^c^	
≥80	16 g per day (in 2 or 4 divided doses) (1 h or 4 h infusion)	8 g per day (in 2 divided doses) (1 h or 4 h infusion)	Initial dosage
50 to <80	12 g per day (in 2 to 3 divided doses) (1 h or 4 h infusion)	6 g per day (in 1 to 3 divided doses) (1 h or 4 h infusion)	Reduce maintenance dosage by 25%
30 to <50	8 g per day (in 2 divided doses) (1 h or 4 h infusion)	4 g per day (in 1 to 2 divided doses) (1 h or 4 h infusion)	Reduce maintenance dosage by 50%
15 to <30	6 g per day (in 1 to 2 divided doses) (1 h or 4 h infusion)	3 g per day (in 1 to 2 divided doses) (1 h or 4 h infusion)	Reduce maintenance dosage by 62.5%
<15	4 g per day (in 1 divided doses) (1 h infusion)	2 g per day (in 1 divided doses) (1 h infusion)	Reduce maintenance dosage by 75%

^a^ Creatinine clearance (CLCr) estimated by Cockcroft-Gault Equation. ^b^ Clinical and Laboratory Standards Institute (CLSI) breakpoints (MICs ≤ 64 mg/L) to treat urinary tract infection caused by carbapenem-resistant *Escherichia coli*. ^c^ European Committee on Antimicrobial Susceptibility Testing (EUCAST) breakpoints (MICs ≤ 32 mg/L) to treat carbapenem-resistant Enterobacterales infection.

**Table 3:** Dosing regimens of intravenous fosfomycin disodium for Monte Carlo simulation in critically ill patients with weights of 50 kg and 70 kg, and various degrees of renal function.

Dosing Regimens for Monte Carlo Simulation		CLCr (mL/min) ^a^				
Daily Doses	Dosing Regimens	≥80	50 to <80	30 to <50	15 to <30	<15
24 g/day	8 g q8h (1 h infusion)	/ ^b^	/ ^c^			
	8 g q8h (4 h infusion)	/ ^c^	/ ^c^			
18 g/day	6 g q8h (1 h infusion)	/ ^b^	/ ^c^			
	6 g q8h (4 h infusion)	/ ^c^				
16 g/day	8 g q12h (1 h infusion)	/ ^b^		/ ^b^		
	8 g q12h (4 h infusion)	/ ^c^	/ ^c^			
	4 g q6h (1 h infusion)	/ ^b^				
	4 g q6h (4 h infusion)	/ ^c^				
12 g/day	6 g q12h (1 h infusion)	/ ^b^	/ ^c^	/ ^b^		
	6 g q12h (4 h infusion)	/ ^c^	/ ^c^			
	4 g q8h (1 h infusion)	/ ^b^	/ ^c^	/ ^b^	/ ^b^	
	4 g q8h (4 h infusion)	/ ^c^	/ ^c^	/ ^c^		
8 g/day	4 g q12h (1 h infusion)	/ ^c^	/ ^c^	/ ^b^	/ ^b^	
	4 g q12h (4 h infusion)	/ ^c^		/ ^c^		
6 g/day	6 g q24h (1 h infusion)		/ ^c^		/^c^	/ ^c^
	6 g q24h (4 h infusion)		/ ^c^	/ ^c^	/ ^c^	/ ^c^
	3 g q12h (1 h infusion)		/ ^c^		/ ^b^	/ ^c^
	3 g q12h (4 h infusion)		/ ^c^			
	2 g q8h (1 h infusion)		/ ^c^			
	2 g q8h (4 h infusion)		/ ^c^			
4 g/day	4 g q24h (1 h infusion)			/ ^c^		/ ^b^
	4 g q24h (4 h infusion)			/ ^c^	/ ^c^	
	2 g q12h (1 h infusion)			/ ^c^		
	2 g q12h (4 h infusion)			/ ^c^		
3 g/day	3 g q24h (1 h infusion)				/ ^c^	/ ^b^
	3 g q24h (4 h infusion)				/ ^c^	
	1.5 g q12h (1 h infusion)				/ ^c^	
2 g/day	2 g q24h (1 h infusion)					/ ^c^

/ = each dosing regimen was based on each CLCr; q6h = every 6 h; q8h = every 8 h; q12h = every 12 h; q24h = every 24 h. ^a^ Creatinine clearance (ClCr) estimated by Cockcroft-Gault Equation. ^b^ Dosage regimens were modified from the manufacturer’s recommendations for intravenous fosfomycin disodium (Nordic Pharma UK Limited, Germany). ^c^ Dosage regimens were determined by our studies.

## Supplementary Materials

The following are available online at https://www.mdpi.com/2079-6382/9/9/615/s1, Table S1: Probability of achieving the AUC_0-24_/MIC of ≥21.5 against carbapenem-resistant Enterobacterales for each intravenous fosfomycin disodium dosing regimens in critically ill patients with various degrees of renal function and weight of 50 kg, Table S2: Probability of achieving the AUC_0-24_/MIC of ≥21.5 against carbapenem-resistant Enterobacterales for each intravenous fosfomycin disodium dosing regimens in critically ill patients with various degrees of renal function and weight of 70 kg.
