# Supplementary material for: Evaluation of Intravenous Fosfomycin Disodium Dosing Regimens in Critically Ill Patients for Treatment of Carbapenem-Resistant Enterobacterales Infections Using Monte Carlo Simulation
Source: Antibiotics (Basel). 2020 Sep 18;9(9):615. doi: 10.3390/antibiotics9090615 (PMC7558518; doi:10.3390/antibiotics9090615)
Supplement: Supplementary file 1 [file antibiotics-09-00615-s001.pdf]

Supplemental Material

Evaluation of Intravenous Fosfomycin Disodium Dosing Regimens in Critically Ill Patients for Treatment of Carbapenem-Resistant Enterobacterales Infections Using Monte Carlo Simulation

Pannee Leelawattanachai [1,2], Thitima Wattanavijitkul [3], Taniya Paiboonvong [4], Rongpong Plongla [5,6], Tanittha Chatsuwan [6,7], Sang Usayaporn [3], Wichit Nosoongnoen [1] and Preecha Montakantikul [1,*]

[1] Division of Clinical Pharmacy, Department of Pharmacy, Faculty of Pharmacy, Mahidol University, Bangkok, Thailand; pannee@nmu.ac.th (P.L.); wichit.nos@mahidol.ac.th (W.N.)

[2] Department of Pharmacy, Faculty of Medicine Vajira Hospital, Navamindradhiraj University, Bangkok, Thailand

[3] Department of Pharmacy Practice, Faculty of Pharmaceutical Sciences, Chulalongkorn University, Pathumwan, Bangkok, Thailand; thitima.w@pharm.chula.ac.th (T.W.); sang.u@pharm.chula.ac.th (S.U.)

[4] Department of Pharmacy Practice, College of Pharmacy, Rangsit University, Pathumthani, Thailand; taniya.p@rsu.ac.th (T.P.)

[5] Division of Infectious Diseases, Department of Medicine, Faculty of Medicine, Chulalongkorn University and King Chulalongkorn Memorial Hospital, Thai Red Cross Society, Bangkok, Thailand; rongpong.p@chula.ac.th (R.P)

[6] Antimicrobial Resistance and Stewardship Research Unit, Chulalongkorn University, Bangkok, Thailand; tanittha.c@chula.ac.th (T.C.)

[7] Department of Microbiology, Faculty of Medicine, Chulalongkorn University, Bangkok, Thailand

[*] Correspondence: preecha.mon@mahidol.ac.th (P.M.); Tel./Fax: (+66) 2-644-8694

Table S1. Probability of achieving the AUC_{0-24}/MIC of ≥ 21.5 against carbapenem-resistant Enterobacterales for each intravenous fosfomycin disodium dosing regimens in critically ill patients with various degrees of renal function and weight of 50 kg.

CLCr (mL/min)	Dosing regimens for simulation with body weights			%PTA for AUC_{0-24}/MIC of ≥ 21.5 by MICs (mg/L)								%CFR	
	Daily doses	Dosing regimens	WT (kg)	32[a]	48[b]	64[c]	96	128[c]	192	256[c]	1024[d]	CRE (n=129)	CR-KP (n=116)
≥80	24 g/day	8 g q8h[e] (1 h infusion)	50	99.98	99.71	99.02	95.95	90.81	70.34	42.46	0	60.97	56.60
		8 g q8h[f] (4 h infusion)	50	99.96	99.65	98.94	95.78	89.45	66.52	35.54	0	60.80	56.40
	18 g/day	6 g q8h[e] (1 h infusion)	50	99.75	99.09	97.52	90.88	78.74	43.29	12.25	0	59.84	55.34
		6 g q8h[f] (4 h infusion)	50	99.78	98.91	96.91	89.45	75.67	35.13	6.21	0	59.58	55.05
	16 g/day	8 g q12h[e] (1 h infusion)	50	99.70	98.65	96.58	88.34	75.43	39.46	11.10	0	59.58	55.04
		8 g q12h[f] (4 h infusion)	50	99.69	98.57	96.50	87.47	72.54	32.98	6.21	0	59.37	54.82
		4 g q6h[e] (1 h infusion)	50	99.73	98.46	96.04	85.47	67.90	23.49	2.65	0	59.06	54.47
		4 g q6h[f] (4 h infusion)	50	99.68	98.27	95.12	82.84	61.39	15.00	0.71	0	58.65	54.02
	12 g/day	6 g q12h[e] (1-h infusion)	50	99.10	96.70	91.96	75.89	52.56	11.09	0.45	0	57.91	53.19
		6 g q12h[f] (4 h infusion)	50	99.12	96.41	91.44	72.62	45.98	6.26	0.07	0	57.56	52.80
		4 g q8h[e] (1 h infusion)	50	99.21	96.18	90.67	71.74	43.40	4.58	0.06	0	57.41	52.63
		4 g q8h[f] (4 h infusion)	50	98.81	95.76	89.35	65.60	34.39	1.64	0.01	0	56.83	51.99
	8 g/day	4 g q12h[f] (1 h infusion)	50	96.68	88.81	75.70	39.10	10.56	0.02	0	0	54.26	49.14
		4 g q12h[f] (4 h infusion)	50	96.47	87.74	73.13	32.39	6.09	0.01	0	0	53.77	48.59
50 to <80	24 g/day	8 g q8h[f] (1 h infusion)	50	100	99.95	99.87	98.93	97.25	87.62	65.36	0	61.71	57.42
		8 g q8h[f] (4 h infusion)	50	100	99.97	99.8	98.99	96.98	84.23	54.83	0	61.50	57.18
	18 g/day	6 g q8h[f] (1 h infusion)	50	100	99.84	99.52	97.41	91.76	64.24	25.14	0	60.82	56.42
	16 g/day	8 g q12h[f] (4 h infusion)	50	99.95	99.74	99.18	96.12	88.48	53.89	14.38	0	60.49	56.06

Table S1. Continued.

CLCr (mL/min)	Dosing regimens for simulation with body weights			%PTA for AUC_{0-24}/MIC of ≥ 21.5 by MICs (mg/L)								%CFR	
	Daily doses	Dosing regimens	WT (kg)	32[a]	48[b]	64[c]	96	128[c]	192	256[c]	1024[d]	CRE (n=129)	CR-KP (n=116)
50 to <80	12 g/day	6 g q12h[f] (1 h infusion)	50	99.84	99.18	97.75	90.17	73.57	22.98	1.47	0	59.45	54.91
		6 g q12h[f] (4 h infusion)	50	99.88	99.19	97.47	88.43	68.32	15.11	0.28	0	59.17	54.59
		4 g q8h[f] (1 h infusion)	50	99.81	99.14	97.26	87.18	65.08	11.42	0.21	0	58.99	54.40
		4 g q8h[f] (4 h infusion)	50	99.82	99.06	96.94	84.31	55.58	5.17	0.03	0	58.56	53.92
	8 g/day	4 g q12h[f] (1 h infusion)	50	99.38	96.56	90.25	61.88	23.71	0.21	0	0	56.55	51.69
	6 g/day	6 g q24h[f] (1 h infusion)	50	98.03	93.11	83.88	51.87	17.10	0.08	0	0	55.49	50.51
		6 g q24h[f] (4 h infusion)	50	97.91	92.54	82.75	46.68	12.47	0	0	0	55.14	50.12
		3 g q12h[f] (1 h infusion)	50	97.67	89.64	73.06	22.55	1.28	0	0	0	53.64	48.45
		3 g q12h[f] (4 h infusion)	50	97.17	87.72	67.09	13.98	0.30	0	0	0	53.01	47.75
		2 g q8h[f] (1 h infusion)	50	96.93	87.15	65.27	12.04	0.24	0	0	0	52.83	47.55
		2 g q8h[f] (4 h infusion)	50	96.45	83.68	55.12	5.10	0.02	0	0	0	52.01	46.64
30 to <50	16 g/day	8 g q12h[e] (1 h infusion)	50	99.98	99.96	99.82	99.25	97.36	80.79	41.14	0	61.31	56.97
	12 g/day	6 g q12h[e] (1 h infusion)	50	99.98	99.87	99.54	97.02	88.99	40.59	3.89	0	60.37	55.93
		4 g q8h[e] (1 h infusion)	50	99.96	99.82	99.49	96.20	82.99	23.43	0.87	0	60.00	55.52
		4 g q8h[f] (4 h infusion)	50	99.96	99.82	99.42	94.60	75.35	11.55	0.10	0	59.63	55.10
	8 g/day	4 g q12h[e] (1 h infusion)	50	99.87	99.35	97.34	79.88	40.60	0.57	0	0	58.00	53.29
		4 g q12h[f] (4 h infusion)	50	99.89	99.22	96.69	73.85	29.15	0.08	0	0	57.46	52.70
	6 g/day	6 g q24h[f] (4 h infusion)	50	99.65	98.29	94.10	68.57	25.91	0.09	0	0	57.05	52.24

Table S1. Continued.

CLCr (mL/min)	Dosing regimens for simulation with body weights			%PTA for AUC_{0-24}/MIC of ≥ 21.5 by MICs (mg/L)								%CFR	
	Daily doses	Dosing regimens	WT (kg)	32[a]	48[b]	64[c]	96	128[c]	192	256[c]	1024[d]	CRE (n=129)	CR-KP (n=116)
30 to <50	4 g/day	4 g q24h[f] (1 h infusion)	50	98.25	91.58	73.71	16.56	0.46	0	0	0	53.68	48.48
		4 g q24h[f] (4 h infusion)	50	98.18	90.80	69.14	10.56	0.05	0	0	0	53.26	48.02
		2 g q12h[f] (1 h infusion)	50	97.42	80.82	40.94	0.77	0	0	0	0	51.25	45.79
		2 g q12h[f] (4 h infusion)	50	96.87	74.43	28.62	0.09	0	0	0	0	50.38	44.82
15 to <30	12 g/day	4 g q8h[e] (1 h infusion)	50	100	99.98	99.98	99.49	94.49	41.80	2.28	0	60.62	56.21
	8 g/day	4 g q12h[e] (1 h infusion)	50	100	99.94	99.68	94.15	63.45	2.06	0	0	59.19	54.62
	6 g/day	6 g q24h[f] (1 h infusion)	50	99.98	99.90	99.26	91.41	57.05	1.36	0	0	58.90	54.30
		6 g q24h[f] (4 h infusion)	50	100	99.91	99.25	88.73	47.68	0.38	0	0	58.55	53.90
		3 g q12h[e] (1 h infusion)	50	99.98	99.71	97.42	63.85	11.20	0	0	0	56.74	51.89
	4 g/day	4 g q24h[f] (4 h infusion)	50	99.88	98.31	88.55	24.56	0.29	0	0	0	55.01	49.97
	3 g/day	3 g q24h[f] (1 h infusion)	50	99.44	91.74	57.89	1.52	0	0	0	0	52.73	47.43
		3 g q24h[f] (4 h infusion)	50	99.10	87.93	46.88	0.39	0	0	0	0	52.00	46.63
		1.5 g q12h[f] (1 h infusion)	50	97.75	64.44	11.47	0	0	0	0	0	49.32	43.64
<15	6 g/day	6 g q24h[f] (1 h infusion)	50	100	100	99.97	98.35	80.02	5.59	0	0	59.85	55.35
		6 g q24h[f] (4 h infusion)	50	100	100	99.99	97.02	71.57	1.8	0	0	59.53	54.99
		3 g q12h[f] (1 h infusion)	50	100	99.99	99.63	81.95	25.39	0	0	0	57.71	52.98
	4 g/day	4 g q24h[e] (1 h infusion)	50	100	99.97	98.38	59.03	5.24	0	0	0	56.50	51.62
	3 g/day	3 g q24h[e] (1 h infusion)	50	99.96	98.47	80.68	5.35	0.01	0	0	0	54.20	49.07

Table S1. Continued.

CLCr (mL/min)	Dosing regimens for simulation with body weights			%PTA for AUC_{0-24}/MIC of ≥ 21.5 by MICs (mg/L)								%CFR	
	Daily doses	Dosing regimens	WT (kg)	32[a]	48[b]	64[c]	96	128[c]	192	256[c]	1024[d]	CRE (n=129)	CR-KP (n=116)
<15	2 g/day	2 g q24h[f] (1 h infusion)	50	98.44	60.78	5.48	0	0	0	0	0	48.97	43.25

CLCr = Creatinine clearance; WT = weight; PTA = probability of target attainment; MICs = minimum inhibitory concentrations; AUC_{0-24}/MIC = area under the plasma drug concentration-time curve from 0 to 24 h over minimum inhibitory concentration; q6h = every 6 hours; q8h = every 8 hours; q12h = every 12 hours; q24h = every 24 hours; CFR = cumulative fraction of response; CRE = carbapenem-resistant Enterobacterales; CR-KP = carbapenem-resistant *Klebsiella pneumoniae*

[a] European Committee on Antimicrobial Susceptibility Testing (EUCAST) breakpoints for fosfomycin, MICs ≤ 32 mg/L as susceptible (S).

[b] MIC for 50% of carbapenem-resistant Enterobacterales clinical isolates (MIC_{50}).

[c] Clinical and Laboratory Standards Institute (CLSI) breakpoints for fosfomycin, MICs ≤ 64 mg/L as susceptible (S), MIC =128 mg/L as intermediate (I), and MICs ≥ 256 mg/L as resistant (R).

[d] MIC for 90% of carbapenem-resistant Enterobacterales clinical isolates (MIC_{90}).

[e] Dosage regimens were modified from manufacturer's recommendations for intravenous fosfomycin disodium (Nordic Pharma UK Limited, Germany).

[f] Dosage regimens were determined by our studies.

Table S2. Probability of achieving the AUC_{0-24}/MIC of ≥ 21.5 against carbapenem-resistant Enterobacterales for each intravenous fosfomycin disodium dosing regimens in critically ill patients with various degrees of renal function and weight of 70 kg.

CLCr (mL/min)	Dosing regimens for simulation with body weights			%PTA for AUC_{0-24}/MIC of ≥ 21.5 by MICs (mg/L)								%CFR	
	Daily doses	Dosing regimens	WT (kg)	32[a]	48[b]	64[c]	96	128[c]	192	256[c]	1024[d]	CRE (n=129)	CR-KP (n=116)
≥80	24 g/day	8 g q8h[e] (1 h infusion)	70	99.95	99.66	99.09	95.66	89.51	65.62	33.32	0	60.77	56.38
		8 g q8h[f] (4 h infusion)	70	99.96	99.66	99.04	95.11	87.58	58.19	23.87	0	60.55	56.13
	18 g/day	6 g q8h[e] (1 h infusion)	70	99.76	99.06	97.21	89.68	75.37	33.31	6.31	0	59.57	55.04
		6 g q8h[f] (4 h infusion)	70	99.77	99.00	97.13	87.86	70.24	24.19	2.55	0	59.27	54.71
	16 g/day	8 g q12h[e] (1 h infusion)	70	99.70	98.62	96.30	87.73	71.90	30.56	5.46	0	59.32	54.77
		8 g q12h[f] (4 h infusion)	70	99.67	98.21	95.75	85.46	67.35	22.88	2.84	0	59.01	54.41
		4 g q6h[e] (1 h infusion)	70	99.70	98.36	95.53	82.95	61.41	14.65	0.82	0	58.68	54.05
		4 g q6h[f] (4 h infusion)	70	99.55	98.10	94.49	79.31	52.64	7.96	0.13	0	58.19	53.51
	12 g/day	6 g q12h[e] (1 h infusion)	70	98.89	96.19	90.69	71.71	44.03	4.98	0.03	0	57.38	52.61
		6 g q12h[f] (4 h infusion)	70	99.00	96.27	90.15	67.54	36.98	2.79	0.01	0	57.06	52.25
		4 g q8h[e] (1 h infusion)	70	98.99	95.99	89.57	65.17	32.72	1.85	0.04	0	56.81	51.97
		4 g q8h[f] (4 h infusion)	70	98.88	95.32	87.58	59.49	24.68	0.54	0	0	56.29	51.39
	8 g/day	4 g q12h[f] (1 h infusion)	70	96.25	86.78	71.35	30.58	5.68	0	0	0	53.57	48.37
		4 g q12h[f] (4 h infusion)	70	96.06	86.29	68.48	22.49	2.44	0	0	0	53.11	47.86
50 to <80	24 g/day	8 g q8h[f] (1 h infusion)	70	99.98	99.93	99.84	98.96	96.59	82.42	52.51	0	61.45	57.13
		8 g q8h[f] (4 h infusion)	70	99.99	99.96	99.87	98.92	96.29	77.98	42.09	0	61.27	56.93
	18 g/day	6 g q8h[f] (1 h infusion)	70	99.98	99.8	99.48	97.04	89.57	52.81	14.39	0	60.56	56.14
	16 g/day	8 g q12h[f] (4 h infusion)	70	99.97	99.71	99.07	95.48	84.58	40.59	6.98	0	60.19	55.73

Table S2. Continued.

CLCr (mL/min)	Dosing regimens for simulation with body weights			%PTA for AUC_{0-24}/MIC of ≥ 21.5 by MICs (mg/L)								%CFR	
	Daily doses	Dosing regimens	WT (kg)	32[a]	48[b]	64[c]	96	128[c]	192	256[c]	1024[d]	CRE (n=129)	CR-KP (n=116)
50 to <80	12 g/day	6 g q12h[f] (1 h infusion)	70	99.89	99.16	97.47	88.24	64.80	13.31	0.48	0	59.04	54.45
		6 g q12h[f] (4 h infusion)	70	99.85	99.07	97.38	85.5	57.43	7.11	0.10	0	58.69	54.06
		4 g q8h[f] (1 h infusion)	70	99.81	99.15	96.84	83.04	52.80	4.85	0.02	0	58.45	53.79
		4 g q8h[f] (4 h infusion)	70	99.81	98.85	95.95	77.20	41.34	1.87	0	0	57.88	53.16
	8 g/day	4 g q12h[f] (1 h infusion)	70	99.33	95.70	87.27	49.53	12.81	0.02	0	0	55.74	50.78
	6 g/day	6 g q24h[f] (1 h infusion)	70	98.08	92.81	80.88	41.21	8.84	0.02	0	0	54.84	49.78
		6 g q24h[f] (4 h infusion)	70	98.05	92.37	79.26	34.82	6.05	0	0	0	54.52	49.42
		3 g q12h[f] (1 h infusion)	70	97.64	87.91	65.17	12.92	0.39	0	0	0	52.96	47.69
		3 g q12h[f] (4 h infusion)	70	97.00	84.99	57.39	6.95	0.05	0	0	0	52.27	46.92
		2 g q8h[f] (1 h infusion)	70	96.77	83.00	52.99	5.20	0.02	0	0	0	51.91	46.52
		2 g q8h[f] (4 h infusion)	70	95.89	77.47	40.99	1.92	0	0	0	0	50.97	45.47
30 to <50	16 g/day	8 g q12h[e] (1 h infusion)	70	100.00	99.97	99.83	99.11	96.06	70.06	24.80	0	61.05	56.68
	12 g/day	6 g q12h[e] (1 h infusion)	70	99.99	99.87	99.42	96.05	82.46	24.03	1.28	0	59.99	55.51
		4 g q8h[e] (1 h infusion)	70	100	99.85	99.36	94.28	72.90	11.39	0.11	0	59.55	55.01
		4 g q8h[f] (4 h infusion)	70	99.99	99.82	99.27	90.04	60.62	4.47	0.01	0	59.01	54.41
	8 g/day	4 g q12h[e] (1 h infusion)	70	99.85	99.09	95.78	68.99	24.09	0.08	0	0	57.14	52.34
		4 g q12h[f] (4 h infusion)	70	99.89	98.94	95.02	59.91	14.90	0.01	0	0	56.61	51.75
	6 g/day	6 g q24h[f] (4 h infusion)	70	99.71	98.08	92.42	55.42	13.36	0.03	0	0	56.28	51.38

Table S2. Continued.

CLCr (mL/min)	Dosing regimens for simulation with body weights			%PTA for AUC_{0-24}/MIC of ≥ 21.5 by MICs (mg/L)								%CFR	
	Daily doses	Dosing regimens	WT (kg)	32[a]	48[b]	64[c]	96	128[c]	192	256[c]	1024[d]	CRE (n=129)	CR-KP (n=116)
30 to <50	4 g/day	4 g q24h[f] (1 h infusion)	70	98.25	88.84	61.27	7.56	0.09	0	0	0	52.76	47.47
		4 g q24h[f] (4 h infusion)	70	98.08	87.33	55.95	4.49	0.03	0	0	0	52.36	47.02
		2 g q12h[f] (1 h infusion)	70	96.09	68.91	24.29	0.11	0	0	0	0	49.86	44.24
		2 g q12h[f] (4 h infusion)	70	94.82	60.22	14.92	0	0	0	0	0	48.97	43.25
15 to <30	12 g/day	4 g q8h[e] (1 h infusion)	70	100	99.99	99.98	98.54	87.32	22.81	0.38	0	60.22	55.76
	8 g/day	4 g q12h[e] (1 h infusion)	70	100	99.93	99.27	85.88	41.88	0.47	0	0	58.30	53.63
	6 g/day	6 g q24h[f] (1 h infusion)	70	99.99	99.76	98.97	82.16	37.38	0.25	0	0	58.05	53.35
		6 g q24h[f] (4 h infusion)	70	99.99	99.79	98.74	77.14	28.09	0.05	0	0	57.64	52.89
		3 g q12h[e] (1 h infusion)	70	99.97	99.39	94.12	41.88	3.44	0	0	0	52.89	50.87
	4 g/day	4 g q24h[f] (4 h infusion)	70	99.72	96.53	76.40	10.64	0.08	0	0	0	54.03	48.88
	3 g/day	3 g q24h[f] (1 h infusion)	70	98.88	83.38	38.15	0.26	0	0	0	0	51.41	45.96
		3 g q24h[f] (4 h infusion)	70	98.55	76.78	27.54	0.07	0	0	0	0	50.62	45.09
		1.5 g q12h[f] (1 h infusion)	70	93.41	42.55	3.91	0	0	0	0	0	47.67	41.80
<15	6 g/day	6 g q24h[f] (1 h infusion)	70	100	100	99.78	93.3	57.93	1.32	0	0	59.00	54.41
		6 g q24h[f] (4 h infusion)	70	100	100	99.67	89.89	47.3	0.37	0	0	58.58	53.94
		3 g q12h[f] (1 h infusion)	70	100	99.88	97.81	60.73	9.36	0	0	0	56.63	51.77
	4 g/day	4 g q24h[e] (1 h infusion)	70	100	99.54	93.16	34.95	1.42	0	0	0	55.56	50.58
	3 g/day	3 g q24h[e] (1 h infusion)	70	99.89	92.99	57.8	1.29	0	0	0	0	52.82	47.54

Table S2. Continued.

CLCr (mL/min)	Dosing regimens for simulation with body weights			%PTA for AUC_{0-24}/MIC of ≥ 21.5 by MICs (mg/L)								%CFR	
	Daily doses	Dosing regimens	WT (kg)	32[a]	48[b]	64[c]	96	128[c]	192	256[c]	1024[d]	CRE (n=129)	CR-KP (n=116)
<15	2 g/day	2 g q24h[f] (1 h infusion)	70	93.42	35.2	1.46	0	0	0	0	0	47.34	41.44

CLCr = Creatinine clearance; WT = weight; PTA = probability of target attainment; MICs = minimum inhibitory concentrations; AUC_{0-24}/MIC = area under the plasma drug concentration-time curve from 0 to 24 h over minimum inhibitory concentration; q6h = every 6 hours; q8h = every 8 hours; q12h = every 12 hours; q24h = every 24 hours; CFR = cumulative fraction of response; CRE = carbapenem-resistant Enterobacterales; CR-KP = carbapenem-resistant *Klebsiella pneumoniae*

[a] European Committee on Antimicrobial Susceptibility Testing (EUCAST) breakpoints for fosfomycin, MICs ≤ 32 mg/L as susceptible (S).

[b] MIC for 50% of carbapenem-resistant Enterobacterales clinical isolates (MIC_{50}).

[c] Clinical and Laboratory Standards Institute (CLSI) breakpoints for fosfomycin, MICs ≤ 64 mg/L as susceptible (S), MIC =128 mg/L as intermediate (I), and MICs ≥ 256 mg/L as resistant (R).

[d] MIC for 90% of carbapenem-resistant Enterobacterales clinical isolates (MIC_{90}).

[e] Dosage regimens were modified from manufacturer's recommendations for intravenous fosfomycin disodium (Nordic Pharma UK Limited, Germany).

[f] Dosage regimens were determined by our studies.
